# Determinants of patients' satisfaction and trust toward healthcare service environment in general practice clinics

**DOI:** 10.3389/fpsyg.2022.856750

**Published:** 2022-07-29

**Authors:** Yun Ai, Muhammad Khalilur Rahman, Md. Shah Newaz, Md. Abu Issa Gazi, Md. Atikur Rahaman, Abdullah Al Mamun, Xia Chen

**Affiliations:** ^1^School of Sociology and Psychology, Central University of Finance and Economics, Beijing, China; ^2^Faculty of Entrepreneurship and Business, Universiti Malaysia Kelantan, Pengkalan Chepa, Malaysia; ^3^Angkasa-Umk Research Academy (AURA), Universiti Malaysia Kelantan, Pengkalan Chepa, Malaysia; ^4^Department of International Business, Independent University Bangladesh, Dhaka, Bangladesh; ^5^School of Management, Jiujiang University, Jiujiang, China; ^6^UKM-Graduate School of Business, Universiti Kebangsaan Malaysia, Bangi, Malaysia

**Keywords:** satisfaction, trust, healthcare service, word-of-mouth, clinic

## Abstract

This study aimed to examine the healthcare service environment, patients' experience, and responses toward healthcare services in private general practice (GP) clinics. Self-administered questionnaires were used for collecting data from 367 respondents with prior experience in visiting the general practice clinics in Malaysia. SmartPLS statistical tool was used to test the underlying hypotheses. The results revealed that ambiance, service delivery, interior decor, and cleanliness had a significant influence on patients' trust and satisfaction while the exterior design is neither associated with satisfaction nor trust. Patients' satisfaction and trust had a higher significant effect on their repatronage intention, willingness to pay for a premium healthcare service, and engagement in word-of-mouth for healthcare services. In practice, both the service delivery and ambiance features of the healthcare services environment might be optimized by GP clinics. This research provides significant insights from the patients' perspective toward the GP clinics' healthcare services environment.

## Introduction

Recent decades have witnessed an array of transformations in the healthcare industry concerning service delivery. These transformations have altered the business or operation of general practice (GP) clinics dramatically. Over time, factors like technology, hyper-competition, mergers of healthcare industry players, rising operation costs, and patient sophistication have put pressure on the health clinic business ecosystem from the outside (Pettigrew et al., 2019; Rahman, 2019; Rahman et al., 2021a). The basic, overly-generalized, and non-specialized characteristics of service subdues the health clinics to be inadequately differentiated, hence, placing them in a competitively disadvantageous situation (Kenny et al., 2017). Thus, the present study looks at the GP clinics' service delivery from a commonly ignored aspect of the “service environment” and demonstrates how it influences patients' experience and responses to provide alternative means for clinics to differentiate their service delivery, and in turn, recoup some competitive advantage. In this study, service environment is associated with ambiance, service delivery, interior decor, exterior design, and cleanliness, whereas patients' experience is related to patients' satisfaction and trust. In addition, their responses are associated with word-of-mouth, repatronage intention, and willingness to pay a premium for healthcare service delivery.

General practice clinic refers to the branch of medicine that provides general, and community-based healthcare services in a personalized manner to patients, families, and friends (Pilnick and Coleman, 2003). GP provides continuous and coordinated care so that the patients' health is monitored in the community. In Malaysia, GP services can be acquired through either public or private channels. The government-owned public primary care centers known as “Klinik Kesihatan” provide GP services at the district level with minimal charges. General practice is characterized as the division of medicine that renders community-based general (MacAllister et al., 2016; Rahman et al., 2021a) and extensive elementary care in a personalized fashion to patients irrespective of the kind of disease or characteristics of the complaint (AFPM, 2016). Likewise, it includes cases that need both critical and specialized care at secondary and tertiary institutions (Rahman et al., 2017). After getting released from hospitals, coordinated and continuous care is provided by these GP clinics (Rahman et al., 2021b) to monitor the patients' health thereafter (Rahman et al., 2018). They accommodate a variety of medical disciplines ranging from dermatology to radiology, neurology, psychiatry, medical, and surgical. Nevertheless, due to inadequate instruments and skill sets, GP healthcare providers are barely able to afford elementary or primary level healthcare.

In Malaysia, physicians with degrees such as M.B.B.S (Bachelor of Medicine or Surgery) and M.D. (Medical Doctorate) from the Malaysian Medical Council (MMC) accredited institutions are allowed to do general practice. The practitioners are inspired to go through professional training to be in sync with the standard practice protocols of the developed countries. Malaysia's general practice clinics are under private ownership. The sector is immensely competitive due to the large number of GP clinics in operation. External challenges like technology and sophisticated medical care service providers, along with internal issues like undifferentiated service are collectively making the GP clinics' business prognosis unattractive (Fong and Kumar, 2017). Furthermore, these challenges threaten the survival of GP clinics as a business model.

In 2017, the number of GP clinics in Malaysia surpassed 7,000 (Thomas, 2017), which inflicts both competitive pressure and threat on GP clinics' business (Teo, 2017). The soaring cost of medical procedures makes general practice services a less attractive investment, especially in the economic downturn the nation currently faces (Teo, 2017; Apergis et al., 2020; Grifka et al., 2022). Additionally increasing operational expense, which includes the cost of drugs, medical devices, rental, legal, and regulatory expenses have hiked GP clinics' fees. The hiked charges have resulted in decreased satisfaction and trust among patients which in turn have reduced their word-of-mouth, willingness to pay a premium, and patronage, given the spiraling cost of living. Against this background, this study explores the GP clinics' healthcare services environment as an alternative means of enhancing patients' experience and their response, in the hope of providing GP clinics with some recommendations to improve their competitive edge.

Patients' responses and experiences in the healthcare service environment have been studied to some extent (MacAllister et al., 2016; Torkzad and Beheshtinia, 2019; Vardaman et al., 2020). For instance, LaVela et al. (2016) mentioned that numerous cross-cutting antecedents contribute toward patient experiences (e.g., satisfaction) based on the atmosphere where the healthcare is obtained. They further urged for high-quality research on the healthcare environment to create a meaningful impact on patients' health outcomes, satisfaction, and overall experience. Most frequently therapeutics, ergonomics, safety, and medical services purposes have been investigated at GP clinics (Stone and McCloy, 2004; Cappelleri et al., 2014). Due to major deviations in the physical settings of different medical specializations and disciplines, the outcomes of previous studies on the healthcare service environment have diverged from one form of healthcare service to another (Han et al., 2018). The study of Rahman et al. (2021d) is the only recent study in the Malaysian healthcare context that addresses the service environment and patients' responses which motivated this study to extend and fill the missing links and enhance the body of knowledge in the healthcare service domain.

This study utilizes a more complex model taking more variables and connections into consideration that were left out by the previous study, and ultimately illustrates a holistic view of the topic in Malaysia. COVID-19 has severely impacted the healthcare service environment in Malaysia and this study would also showcase the changes (if any) that have been influencing the Malaysian healthcare service in a post-covid world. Therefore, the physical attributes examined by those research differ which begs the unanswered question of whether the findings could be applied to GP clinics, particularly, in the context of Malaysia which is yet to be verified. Therefore, this study analyzes the influence of general practice clinic healthcare services environment (e.g., ambiance, exterior design, cleanliness and interior decor, and service delivery) on patient experience (e.g., satisfaction and trust), which in turn inspire patient positive responses (e.g., word-of-mouth, repatronage, and willingness to pay a premium). The results would provide marketing and commercial propositions for the private GP clinics' struggling to survive.

## Literature review

The ambiance of a place is characterized by sensorial environmental stimulations such as lighting, fragrance, temperature, and background sound. These stimulants, for specific hospitality sectors like hotels and restaurants, can be powerful for customer response and experience (Hooper et al., 2013; Jamshidi et al., 2020). Overall, the influence of these features on the service environment varies from sector to sector based on the types of customers. Horng et al. (2013) reported that ambiance influences satisfaction and trust in a restaurant setting. The underlying conditions of ambiance possess high predictive power when it comes to the positive response of the patients' experience toward service delivery (Liu and Jang, 2009) and play a crucial role in customer responses (Jani and Han, 2015). In retail stores, music and rhythm influence the length of stay, customer experience, and amount of money spent (Rahman et al., 2021b); soft lighting, classic music, and thick carpeting impact customers' perception of merchandise quality (Spangenberg et al., 2006); and fragrance, particularly vanilla, are preferred by customers (Morrison, 2011). Ladhari et al. (2017) postulated that ambiance is a crucial component of customers' experience among fashion wear retailers. This study examines how GP clinics' ambiance influences patients' satisfaction and positive responses. Thus, we postulated that:

H1a: Ambiance has a significant influence on patients' satisfaction with healthcare service delivery.
H1b: Ambiance has a significant influence on patients' trust toward healthcare service delivery.

Aesthetic features of the clinic's exterior such as the name, signage board, and glass panel design, also constitute the hedonic components of the clinic's service atmosphere. Symbols and signs serve both symbolic and functional objectives. Hooper et al. (2013) stated that the utilitarian purpose of navigation is served by the direction signage panel, while the logo and brand name are relevant at the symbolic level. Day (2020) explained the link between risk management and exterior wall systems and identified that a durable design and construction of exterior walls can be achieved. Mukhopadhyaya et al. (2020) stated that the exterior walls are designed for optimum moisture management. Rahman (2019) posited that patients are usually prompt to obtain healthcare services delivery at clinics with attractive exterior designs. Accordingly, healthcare institutions usually invest huge resources for a satisfactory service environment to obtain patients' trust and provide better satisfaction. According to Ramli (2019), and Gong et al. (2020), for an appealing design, elements such as the lighting, color, and layout are the most essential for patients' trust and satisfaction. Therefore, we formulated the following hypotheses.

H2a: Exterior design is positively related to patients' satisfaction with GP clinics.
H2b: Exterior design is positively related to patients' trust in GP clinics.

Interior décor includes displays (posters and paintings), as well as wall, floor styling, and furniture. It further emphasizes the hedonic components of the service atmosphere. Gong et al. (2020) postulated that the wallpaper design, paintings, and interior décor of the service environment symbolically communicate a sensation of style. Similarly, Hooper et al. (2013) believed that interior décor provided a sense of style. Mahajan et al. (2021) stated that a good design service can influence customers' trust and satisfaction. Kang (2018) explained the significance of how fashion and aesthetics have a bearing on social life since fashion presents the aesthetic quality as well as the social ties. Interior décor, in the context of the healthcare environment, also includes functional components such as the waiting room, reception counter for the ease of patient navigation, aisles and corridors, and a consultation room (Han et al., 2018). The interior design of the clinics may influence patients' different feelings, experiences, and trust. Accordingly, we suggest the following hypotheses:

H3a: Interior decor is positively related to patients' satisfaction with GP clinics.
H3b: Interior decor is positively related to patients' trust in GP clinics.

Cleanliness refers to the hygiene related to the service environment like the reception area, sidewalk in front of the clinic, consultation room, waiting area, and staff attire. Awan et al. (2020) focused on the level of cleanliness in the hotel industry during the pandemic, and the result indicated that cleanliness was a crucial factor that influenced customers' trust and satisfaction. Cleanliness or hygiene factors are taken as physical dimensions of the service atmosphere which were perceived to influence satisfaction and trust in both the emergency department and outpatient settings (Akmaz and Çadirci, 2017). Previous studies have shown the importance of cleanliness on satisfaction and trust (Mona et al., 2014; Ferreira et al., 2018) in healthcare providers where the focus has been on cleanliness in washrooms and surrounding areas of the hospital for the sake of the safety and contentment of the patients (Rahman, 2019; Giusti et al., 2020; Rahman et al., 2021c). Clean waiting areas and wards can influence the satisfaction of patients and their confidence in healthcare service. Javed et al. (2021) identified cleanliness to influence patients' satisfaction in the emergency department setting. Based on these indicators, we propose that:

H4a: Cleanliness positively influences patients' satisfaction with healthcare service delivery in GP clinics.
H4b: Cleanliness positively influences patients' trust in healthcare service delivery in GP clinics.

Service delivery refers to the social aspects of the physical environment (Jessup et al., 2020) including service mannerisms such as empathy, friendliness, and kindness toward the patients (Upadhyai et al., 2020). It is evaluated differently compared to other service environments like restaurants, banks, or airlines. This happens because it is difficult for the patients to determine or evaluate the technical characteristics of services that medical facilities offer (Sahoo and Ghosh, 2016). The service delivery construct includes generic service elements such as waiting time, patients' comfort, and a calm environment. Social interaction within the service environment is taken into consideration (Han et al., 2018). In this study, patients' waiting time, as well as doctors' and clinic personnel's capacity to interpret medical reports and exhibit caring, friendly, and generous attitudes are assessed. According to Sahoo and Ghosh (2016), healthcare service settings determine patients' satisfaction and trust. In this context, Akmaz and Çadirci (2017) examined the outpatient hospitals' healthcare services on satisfaction. This study postulates the following hypotheses:

H5a: Service delivery positively influences patients' satisfaction with healthcare service delivery in GP clinics.
H5b: Service delivery positively influences patients' trust in healthcare service delivery in GP clinics.

Satisfaction can be defined as an adequate sensation of joy or comfort and the resulting fulfillment that originates from cognitive evaluations of thoughts, experiences, and events (Wong, 2004). From this, one could deduce that satisfaction and trust have both affective and cognitive components. If the service performance meets their expectation, the patients would be satisfied and it is reflected in their word-of-mouth, repatronage intention, and willingness to pay a premium for healthcare services. If the service performance is below expectations, the patients would be unsatisfied. Ladhari (2009) summarized the cognitive and affective components of satisfaction. While cognitive satisfaction is related to patients' rational judgment of service quality and environment, satisfaction is patients' affective or emotional expression such as pleasure, trust, happiness, and joy upon service encounter. The hospital service environment can influence patient satisfaction and in turn reflect patients' word-of-mouth (Coutinho et al., 2020), repatronage intention (Odoom et al., 2021), and willingness to pay a premium (Batbold and Pu, 2021) toward healthcare service delivery. In their study, Rahman et al. (2021b) explained the impact of patients' satisfaction and patients' responses toward healthcare services at general practice clinics. Therefore, we postulate the following hypotheses:

H6a: Satisfaction has a significant influence on word-of-mouth toward healthcare service delivery in GP clinics.
H6b: Satisfaction has a significant impact on repatronage intention toward healthcare service delivery in GP clinics.
H6c: Satisfaction has a significant influence on the willingness to pay a premium toward healthcare service delivery in GP clinics.

Trust has been widely investigated in various fields of study such as leadership (Dirks and Ferrin, 2002), information technology, and even specialized microsurgical techniques (Abbott et al., 2003). In the service industry, trust is defined as the perception of confidence, belief, and reliance of a person toward another party's intention to do well and cause no harm to the person's interest (Rotenberg, 2001). Hall et al. (2002) clarified interpersonal trust in the primary healthcare setting and explained the physicians' ability to communicate medical information and demonstrate medical competence in addition to developing a rapport, being empathetic, and caring. To measure patients' trust in GP clinics' service environment, this study employs a more tangible assessment using the trust scale (Koschate-Fischer and Gartner, 2015). The scale not only measures the organization's performance, but involves other multi-dimensional factors such as word-of-mouth, repatronage intention, and willingness to pay the premium, which are the measures for this study. Word-of-mouth has demonstrated its influence in the virtual realm. Patients' positive word-of-mouth is the outcome of the affirmative emotional experience of the service encounter. In the healthcare setting, Akmaz and Çadirci (2017) showed repatronage intention among emergency department patients. Ladhari (2009) stated that patients are willing to pay more in a preferable service environment when they trust the service providers. Patients' willingness to pay a premium for the clinic service environment is based on their trust in the overall service environment. Given this assessment, we suggest the following hypotheses:

H7a: Trust has a significant influence on word-of-mouth toward general practice clinics.
H7b: Trust has a significant influence on repatronage intention toward general practice clinics.
H7c: Trust has a significant influence on the willingness to pay a premium for general practice clinics.

In the healthcare service sector, patients' satisfaction is a vital measurement of performance, particularly, in the context of developing economies (Alrubaiee and Alkaa'ida, 2011). A recent study in telemedicine posits that patients tend to put their trust in the medical service providers which is one of the building blocks for promoting patient satisfaction, hence, a higher trust is found to be associated with higher patient satisfaction (Orrange et al., 2021). The seminal work of Platonova et al. (2008) also validates that patient trust and good interpersonal relationships are major predictors of satisfaction. Therefore, patients' trust in the healthcare service delivery can reflect patients' satisfaction (Alrubaiee and Alkaa'ida, 2011). Hence, this study proposes the below hypothesis.

H8: There is a significant relationship between trust and satisfaction toward general practice clinics.

In the healthcare paradigm, the acquired services hugely rely on patients' experience (Blut et al., 2014; Lee et al., 2020), just as in their satisfaction and trust. In the prior sections, this study established with the extant literature that several factors stemming from the service environment literature such as ambiance, service design (exterior and interior), cleanliness, and service delivery aspects greatly influence both the trust and satisfaction of the patients. For instance, ambiance was found influential in settings such as restaurants (Horng et al., 2013), music shops (Jani and Han, 2015), and retail stores (Ladhari et al., 2017) that enhance or diminish customer responses in terms of trust or satisfaction. When it comes to exterior and interior design, the appeal of the design influences patients' trust and satisfaction (Gong et al., 2020). Mahajan et al. (2021) noted that a well-designed service can gain patients' trust and satisfaction, while Awan et al. (2020) flagged cleanliness as a critical enabler that influenced patient trust and satisfaction in the hotel service. Though it is difficult for patients to evaluate the technical aspects of healthcare services, Sahoo and Ghosh (2016) noted service environment as a critical determinant of patients' satisfaction and trust.

Furthermore, patient “satisfaction” which is one of many aspects of the patients' experience reflect word-of-mouth (Coutinho et al., 2020), repatronage intention (Odoom et al., 2021), and willingness to pay a premium (Batbold and Pu, 2021) toward healthcare service delivery. “Trust” is another factor that is considered by this study to incorporate patients' experience and has been proven to impact repatronage intention among emergency department patients (Akmaz and Çadirci, 2017) and patients are also willing to pay more for a preferable service environment (Ladhari, 2009). Summing up all these associations, the set of factors that have emerged in the context of the healthcare service environment are ambiance, exterior design, interior decor, cleanliness, and service delivery. These factors ultimately impact the WOM, willingness to pay a premium, and repatronage intention of patients based on their satisfaction and trust. This makes trust and satisfaction play the mediating role in these relationships. Thus, the following two hypotheses are proposed.

H9: Satisfaction mediates the relationship between independent factors (ambiance, exterior design, interior decor, cleanliness, and service delivery) and dependent factors (word-of-mouth, repatronage intention, and willingness to pay a premium).
H10: Trust mediates the relationship between independent factors (ambiance, exterior design, interior decor, cleanliness, and service delivery) and dependent factors (word-of-mouth, repatronage intention, and willingness to pay a premium).

This study bases its assumptions on the Theory of Environmental Psychology (Mehrabian and Russell, 1974) to assess the service environment of healthcare clinics, patient satisfaction and trust, as well as patients' response that includes a willingness to pay a premium, word-of-mouth, and repatronage intention. As patients would not possess any physical ownership of merchandise in any service-related transaction, the assessment of the availed healthcare service heavily relies on patients' satisfaction (Selim et al., 2019). Likewise, since the benefits gained are typically intangible, the service providers' performance too heavily hinges on the patients' satisfaction during the service encounter (Blut et al., 2014; Lee et al., 2020). Hence, the healthcare services environment gains importance as it shapes patient satisfaction and trust.

**Figure 1:**
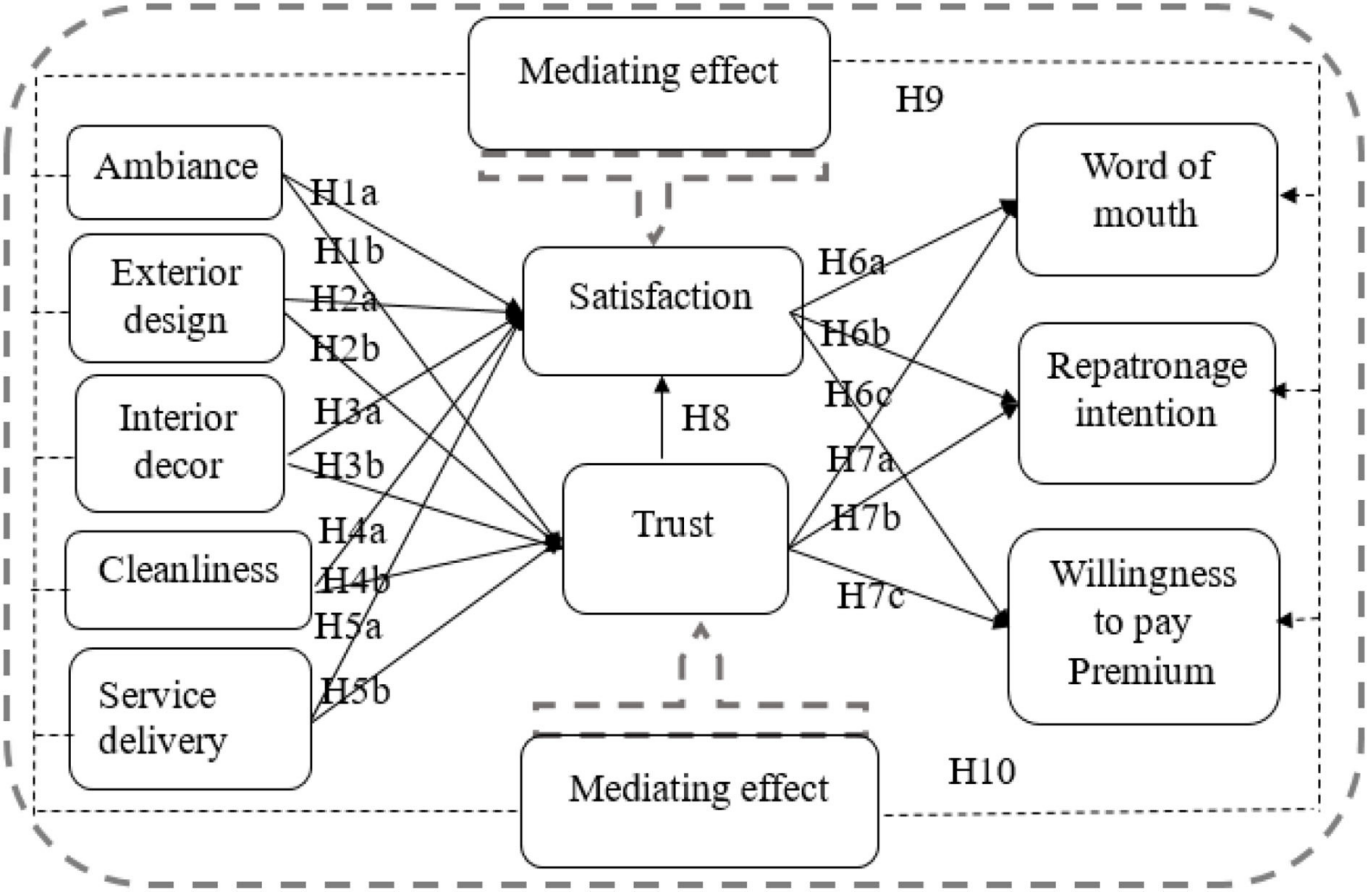
Conceptual model.

This study provides a holistic view of how the GP clinics' healthcare service environment (ambiance, exterior design, cleanliness, interior decor, and service delivery) may influence the patient experience (in terms of satisfaction and trust), and how these can be associated to inspire patient positive responses. Following the abovementioned literature review and underpinning theoretical discussion, this study formulates the conceptual model as presented in Figure 1.

## Methodology

### Measurement instrument

A questionnaire was developed to meet research objectives and measure relationships among variables in the research framework by adopting items, which were previously validated from several studies. Twenty measurement items were adapted to evaluate the healthcare services environment factors (ambiance, exterior design, interior décor, cleanliness, and service delivery) from the studies of Bitner (1992) and Han et al. (2018) to fit into the GP clinics' service environment in Malaysia. Patients' experiences comprised of two mediating variables, namely; satisfaction and trust. Seven items for patients' satisfaction were adopted from Mehrabian and Russell's (1974). PAD model of environmental psychology, while the five measurement items used for trust were adapted from Koschate-Fischer and Gartner (2015). The patients' response consisted of three dependent variables, namely; willingness to pay a premium, engage in word-of-mouth, and repatronage intention. For word-of-mouth, five measurement items were adopted from Eisingerich et al. (2015), for willingness to pay a premium, four items from Netemeyer et al. (2004). CBBE brand equity elements measurements, and for repatronage intention four measurement items were modified from Grace and O'Cass (2005). A five-point Likert scale from 1 (strongly disagree) to 5 (strongly agree) was used to obtain the respondents' responses in this study.

This study employed the deductive approach and utilized quantitative, cross-sectional, non-experimental techniques to examine the relationships between variables. The SmartPLS 3.2.8 software was used to evaluate the model of this study. The partial least square method was chosen due to its suitability for the model fit without any difficulty (Hair et al., 2019). We used several steps for data analysis following Henseler and Chin's (2010) PLS analysis. A step-by-step PLS analysis procedure was used to estimate the measurement model of the study. As a first step, a reflective model using PLS-SEM was used to evaluate the measurement model (Chin, 1998; Hair et al., 2016) as per the guiding principles to estimate model results (Hair et al., 2019).

### Sampling method and data collection

The sample frame of this study included all patients aged 18 years and above, who had visited a private general practice (GP) clinic. For the data collected from the Malaysian GP clinics, a purposive sampling method was employed. Potential respondents were purposively selected across age groups, gender, and ethnicity to reflect diverse backgrounds and demographics. Participants who consented responded to the survey questionnaire that was provided. Medical professionals were excluded to circumvent any sampling bias. The information regarding the study as well as its purpose was shared. The confidentiality and anonymity of the respondents were emphasized. No incentive was promised. As it was a paper-based face-to-face survey, consent was first taken then the respondents were briefed and instructed thoroughly to fill out the questionnaire.

A pre-test was conducted before the data collection and five opinions were taken from experts well versed in healthcare services. The aim was to test the questionnaire with a smaller sample size to avoid any potential issues before the actual data collection. Feedback from these experts' regarding impressions, understanding, complexity, and clarity, along with the order of the questions, were considered. The pre-test ensured face validity, as well as the criterion validity of the questionnaire (Kimberlin and Winterstein, 2008). Twenty-five participants were recruited for a pilot test. The pilot test was conducted using paper-based questionnaires and in person. The respondents were prompted to examine the questions and contribute with constructive feedback while answering the questionnaire. They were further inspired to write notes on the questionnaire if necessary.

We examined the pre-test and pilot test to take notes on their opinions, comments, and feedback. After completing these tests, a total of 500 questionnaires were distributed (between October and December 2019), and 367 responses were received. Of these, six responses were discarded as they were not in Malaysia and one was discarded because it was incomplete. Altogether, with a response rate of 72%, 360 valid and consistent replies were gathered for the data analysis. G^*^Power software was employed to validate the sample size of this study showing the adequacy of the 360 samples as the sample size. The output of G^*^power registered a significant level of 0.05 and yielding strength of 0.99, symbolizing the satisfactory level of sample power in this study (Chin et al., 2003).

### Common method variance

The common method bias is generated by common method variance. This study used Harman's single factor test to reduce the common method bias. Harman's single-factor test confirmed that there was no common method variance as the highest factor accounted for only 28.15% of the variance, which is lower than the threshold of 50% (Podsakoff et al., 2012). The study reduced the social desirability bias (SDB) which denotes the respondent's tendency to be influenced when filling in the questionnaire while being viewed by others, which can affect the questionnaire's validity (Nederhof, 1985). To reduce SDB for this study, we used three different ways to ensure obscurity, promising privacy, and requesting to be straightforward. First, the respondents in this study did not reveal their names, titles, and name of the company in the survey. The degree of SDB differs from the level of anonymity in the survey. More privacy and trustworthiness are assured which leads to identifying fewer SDBs (Randall and Fernandes, 1991). Second, this investigation maintained confidentiality and the survey used the exploratory results for academic purposes only. Third, the respondents were requested to fill in the survey sincerely. Consequently, there was no SDB in this study.

## Findings

### Demographic analysis

The respondent's demographic information shows that a majority of the respondents (65%) were female; male respondents were about one-third (35%) of the sample. The respondents were mostly Malay (76.3%) and Chinese (17.7%) populations together incorporating around 85% of the sample size. The remaining 5.3% were Indians and 0.7% were from other ethnic groups. More than half of the respondents (57.7%) were married, (36.6%) were single, and 5.6% were divorced and widowed. Age-wise, the sample was evenly distributed; more than 90% of the respondents were from the three middle-aged categories of 21 to 30 years, 31 to 40 years, and 41 to 50 years.

Education-wise, a majority of the respondents were well educated with 45.4% holding a bachelor's degree, followed by master's and professional degree holders with 21.3 and 20.8% respectively. About 10.5% had a high school education, and 2% had a Ph.D. In the sample, 5.3% were unemployed, and 9.7% were not part of the main workforce. A majority of the respondents worked in the private sector (52.4%), followed by 19.4% in the government and 13.2% were self-employed. Finally, the data showed that the majority of the respondents who visit GP clinics came from middle-income to upper-middle-income backgrounds with 30.8% within an income range of RM 2,501 to RM 5,000 (middle class) and 19.6% within an income range of RM 5,001 to RM 10,000 (upper-middle class). About 19.5% made below RM 2500 each month, and 16.2% belong to the upper-income background with a monthly income above RM 10,000 (Table 1).

**Table 1:** Demographic profile.

**Variable**	**Category**	**%**	**Variable**	**Category**	**%**
Gender	Male	35.1	Ethnicity	Malay	76.3
	Female	64.9		Chinese	17.7
Marital status	Single	36.6		Indian	5.3
	Married	57.7		Others	0.7
	Widowed	1.2	Level of Education	High School	10.5
	Divorced	4.4		Professional Certification	20.8
Age	18–20 years old	2.5		Bachelor's Degree	45.4
	21–30 years old	23.1		Masters' Degree	21.3
	31–40 years old	38.9		Doctorate	2.0
	41–50 years old	28.6			
	51–60 years old	6.1	Occupation	Unemployed	5.3
	61 years old and above	0.8		Student	9.7
Monthly income	None	13.9		Self-employed	13.2
	RM 1 to 2,500	19.5		Government Employee	19.4
	RM 2,501 to 5,000	30.8		Private employee	52.4
	RM 5,001 to 10,000	19.6			
	Above RM 10,000	16.2			

Table 2 shows the findings of the respondents' visits to the private general practice clinic.

**Table 2:** Respondents' GP clinic profile.

**Variable**	**Category**	**Percent (%)**	**Variable**	**Category**	**Percent (%)**
Type of clinic	Chain clinic	46.7	How long have the respondents been visiting the clinic	<6 months	29.7
	Sole proprietor	53.3		6 months−1 year	18.9
Clinic location	City center	38.1		1–3 years	14.2
	Suburban residential area	57.5		3–5 years	8.6
	Rural area	4.4		> 5 years	28.6
Reasons for choosing the clinic	Close to home/residence	42.2	Number of visits in the past year	1–2 times	53.1
	The GP has a good reputation	17.5		3–4 times	27.8
	The GP is my family doctor	4.7		5–6 times	8.9
	Company's panel clinic	27.8		> 6 times	10.3
	Insurance's panel clinic	5.6			
	Others	2.2	Perception of overall health	Poor	1.1
Reasons for visiting the clinic	Mild illness	88.6		Fair	27.8
	Longstanding illness	4.7		Good	53.9
	Beauty/ aesthetics	0.8		Very good	15.6
	Pregnancy-related	0.6		Excellent	1.7
	Companionship/ counseling	1.7			
	Others	3.6			

A majority of the respondents (42.2%) chose private GP clinics due to the proximity to their residences, while 27.8% visited them because the clinics were empaneled with the companies they work for. About 17.5% chose particular clinics due to their reputation, and 5.6% were because the clinics were empaneled with their health insurance service provider. Decisions of 4.7% were because the GPs were their family physicians, and the remaining 2.2% stated other reasons such as nearness to the workplace, the GP being a friend, the GP has a good bedside manner, by chance, comfort, and the clinics' availability on public holidays. An overwhelming majority (88.6%) chose mild illnesses as the reason for their GP clinic visits, 4.7% were due to long-standing illnesses, 1.7% were for companionship and counseling, while less than 1% chose aesthetics and pregnancy as the reasons for their GP clinic visits. Around 3.6% stated other reasons including vaccinations, dermatological and gynecological issues, children's circumcision, and competitive fees.

The study's findings revealed that a majority of the respondents were either relatively new to the clinic or had been visiting it for <6 months (29.8%). An equally good number were visiting the clinic on a long-term basis of >5 years (28.6%). About 18.9%, had been visiting between 6 months and 1 year, 14.2% for 1–3 years, and 8.6% between 2 and 5 years. With regard to frequency of visit, 53.1% visited the clinics one to two times, 27.8% three to four times, 8.9% five to six times, and 10.3% visited the clinics more than six times. Finally, a majority of the respondents (53.9%) self-rated their overall health as good, followed by 27.8% as fair, 15.6% as very good, 1.7% as excellent, and 1.1% as poor (Table 2).

Table 3 shows the results of the reliability analysis. Standard deviation on the other hand measures the span of observed values. To numerically assess normality, skewness and kurtosis were the chosen tests of this study. In this study, the skewness value was between −1.5 to 1.5 and the kurtosis value was between −2.0 and 2.0 and was considered within the range of normality (Sheridan and Coakes, 2011). The results indicated that the score of each measuring item of the variables was within the acceptable skewness and kurtosis ranges, which indicated normality.

**Table 3:** Reliability assessment.

**Characteristic**	**Mean**	**SD**	**Skew**.	**Kurt**.	**FL**	**VIF**
**Ambiance**						
The temperature of this clinic is acceptable (AM1)	3.7809	0.63448	−0.447	0.982	0.814	2.300
The lighting was relaxing (AM2)	3.6882	0.73999	−0.436	0.514	0.866	1.849
The atmosphere of this clinic is pleasant (AM3)	3.5674	0.81428	−0.124	−0.157	0.875	1.818
The surrounding sound is peaceful (AM4)	3.5590	0.80799	−0.257	0.071	0.833	1.637
Overall, this clinic's ambiance is cheering (AM5)	3.7360	0.77128	−0.248	−0.050	0.903	1.497
**Exterior design**						
The signage board of this clinic is attractive (ED2)	3.3652	0.90776	0.173	−0.247	0.874	2.241
The clinic name is appealing (ED3)	3.3624	0.84296	0.029	0.147	0.889	2.205
This clinic's exterior design is beautiful (ED5)	3.5000	0.87372	0.268	−0.089	0.852	1.765
**Interior decor**						
The wall color/ wall paper design is pleasing (ID1)	3.3680	0.88937	−0.019	0.144	0.893	1.363
The flooring is attractive (ID2)	3.1910	0.82737	0.201	0.142	0.893	1.199
The furniture is stylish (ID3)	3.0253	0.94463	0.111	0.174	0.867	1.786
This clinic's displays such as pictures, paintings, and posters are appealing (ID4)	3.3062	0.92482	0.021	0.300	0.881	1.904
The decoration of this clinic is eye-catching (ID5)	3.3680	0.88937	−0.019	0.144	0.896	1.217
**Cleanliness**						
The front side of this clinic is clean (CL1)	3.7444	0.79388	0.461	0.108	0.811	2.090
The waiting area is clean (CL3)	3.9551	0.69865	0.487	0.791	3.955	1.527
The consultation room is clean (CL4)	4.0646	0.64023	0.252	0.135	4.064	1.432
The staff's attire is clean (CL5)	4.0028	0.66924	0.230	−0.065	4.002	1.065
**Service delivery**						
The record-keeping personnel of the information desk is friendly (SD1)	3.6938	0.81124	0.245	−0.380	0.765	1.858
The physician's explanation about the medical checkup is clear (SD3)	3.9719	0.78672	0.474	−0.110	0.879	1.044
The doctor is caring toward me (SD4)	3.9916	0.77091	0.357	−0.357	0.893	1.286
The nurses of this clinic is very kind to me (SD5)	3.7837	0.77341	0.341	−0.139	0.878	1.644
**Satisfaction**						
Displeased–Pleased (SA1)	3.7331	0.88755	−0.107	−0.811	0.919	1.810
Frustrating–Enjoyable (SA4)	3.5309	0.85689	0.174	−0.400	0.888	2.049
Unsatisfied–Satisfied (SA5)	3.8287	0.91705	−0.425	−0.414	0.925	1.455
Unwanted–Welcomed (SA7)	3.7893	0.91498	−0.347	−0.483	0.906	1.847
**Trust**						
I am sure that my personal information is kept confidential by the clinic (TR1)	3.8933	0.67957	0.136	−0.200	0.780	1.781
I am confident with the performance of this clinic (TR2)	3.9494	0.68194	0.312	0.159	0.905	1.171
I expect the clinic to deliver its promise (TR3)	3.9326	0.74754	0.378	0.163	0.919	2.183
I trust the clinic (TR4)	3.9129	0.70870	0.113	−0.437	0.931	1.851
**Word-of-mouth**						
I will tell people positive things about this clinic (WM1)	3.8483	0.69162	−0.201	−0.069	0.884	1.069
I will encourage my relatives and friends to take medical treatment in this clinic (WM2)	3.7612	0.76306	−0.256	−0.213	0.898	1.209
I will give a positive review about this clinic on social media (WM3)	3.5590	0.83542	−0.100	−0.120	0.858	2.627
I will recommend this clinic for medical treatment to others on social media (WM5)	3.2500	0.84044	0.016	−0.096	0.763	2.047
**Repatronage intention**						
I am likely to visit this clinic in future (RP1)	3.9101	0.70236	0.462	0.708	0.889	1.881
I see myself revisiting this clinic for my next health check-up (RP2)	3.7753	0.78726	0.486	0.191	0.912	1.707
This clinic will be my first choice for my next health examination (RM3)	3.5730	0.85432	0.202	−0.439	0.898	1.432
I have every intention of visiting this clinic in future (RP4)	3.7191	0.77987	0.323	0.001	0.898	1.040
**Willingness to pay a premium**						
I don't mind paying extra for a reputable clinic (WP1)	3.5421	0.88869	−0.370	0.331	0.813	1.796
I am willing to pay an expensive fee for this clinic (WP2)	3.0787	0.95209	0.118	0.471	0.934	1.479
I will pay more for this clinic than other GP clinics (WP3)	3.0365	0.95316	0.104	0.472	0.931	1.328

For the examination of indicator loadings, factor loadings above 0.708 are recommended. In our study, the factor loadings (FL) ranged from 0.763 to 0.934 pointing out that these constructs have more than 50% explanatory power; which provides an acceptable range for reliability (Hair et al., 2019). Multicollinearity is a situation where there are two or more independent variables that are highly correlated. For multiple regression results to be of value, the data set should not have multicollinearity. This can be evaluated statistically by looking at the variance inflation factor (VIF), and a VIF value of <10 indicates the absence of multicollinearity. In our study, the VIF scores ranged between 1.04 to 2.627 (Table 3) which was below the cut-off point of 3.00, signifying no presence of collinearity amidst the predictor constructs (Becker et al., 2015).

### Measurement model assessment

**Figure 2:**
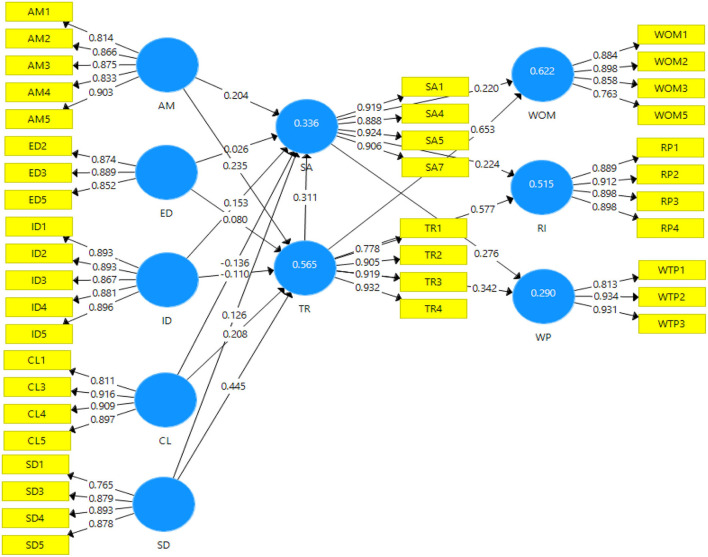
Measurement model.

The findings of the measurement model reveal that composite reliability (CR) and internal consistency reliability had been assessed. The CR values ranged from 0.905 to 0.950 (Table 3) which met the criterion of good reliability since the threshold is from 0.70 and 0.90 (Hair et al., 2019). Though some contemporary researchers take Cronbach's alpha to be a less accurate standard of reliability (Hair et al., 2019); however, it does measure internal consistency reliably. In this study, the alpha values ranged from 0.843 to 0.932 which is above the threshold level of 0.70, hence, registering a higher-bound estimate of reliability (Gefen et al., 2000). To wrap up reliability analysis, the values of rho_A were also considered, which were greater than the cut-off point of 0.70 ranging from 0.847 to 0.935, good enough to show composite reliability (Dijkstra and Henseler, 2015). Third, the convergent validity was measured for the constructs; where average variance extracted (AVE) was applied to evaluate convergent validity (CV) (Hair et al., 2019). According to Fornell and Larcker (1981), the cut-off value for AVE should be above 0.50. Here, AVE values ranged from 0.726 to 0.827 (Table 4) illustrating that the underlying factors of the model had more than 50% explanatory power over the variance of its items. Figure 2 shows the measurement model analysis.

**Table 4:** Convergent validity.

**Constructs and items**	**Cronbach's alpha**	**rho_A**	**Composite reliability**	**Average variance extracted (AVE)**
Ambiance (AM)	0.911	0.915	0.933	0.737
Cleanliness (CL)	0.906	0.914	0.935	0.782
Exterior Design (ED)	0.843	0.847	0.905	0.760
Satisfaction (SA)	0.930	0.932	0.950	0.827
Interior Décor (ID)	0.932	0.935	0.948	0.785
Repatronage Intention RI	0.921	0.922	0.944	0.809
Service Delivery (SD)	0.877	0.885	0.916	0.731
Trust (TR)	0.907	0.918	0.935	0.785
Word-of-mouth (WM)	0.875	0.894	0.914	0.726
Willingness to pay a premium (WP)	0.875	0.903	0.923	0.800

By applying Heterotrait-monotrait (HTMT) ratio and Fornell-Larcker criterion the discriminant validity was assessed. Table 5 explains the correlations between the square root of AVE values and the latent variables that were summarized. The diagonal values of each AVE (square root) were higher than the respective correlation among the latent variable scores presented in the corresponding column and row, showing adequate levels of discriminant validity (Fornell and Larcker, 1981; Hair et al., 2019). Furthermore, Henseler et al. (2015) had suggested HTMT as a solid alternative for Fornell-Larcker; hence, the HTMT ratio was also employed for this study. The HTMT scores ranged from 0.386 to 0.843 (see Table 5), which is below the cut-off point of 0.90, indicating the presence of discriminant validity, in other words, the variables are quite distinct from each other (Henseler et al., 2015; Hair et al., 2019).

**Table 5:** Discriminant validity.

	**AM**	**CL**	**ED**	**ID**	**RI**	**SA**	**SD**	**TR**	**WOM**	**WP**
Fornell-Larcker Criterion										
AM	0.859									
CL	0.704	0.884								
ED	0.561	0.525	0.872							
ID	0.641	0.553	0.694	0.886						
RI	0.589	0.514	0.366	0.418	0.899					
SA	0.485	0.370	0.345	0.409	0.519	0.909				
SD	0.584	0.587	0.359	0.446	0.609	0.455	0.855			
TR	0.616	0.616	0.405	0.410	0.691	0.511	0.684	0.886		
WOM	0.664	0.613	0.511	0.568	0.727	0.554	0.670	0.766	0.852	
WP	0.438	0.381	0.345	0.383	0.525	0.451	0.411	0.484	0.533	0.894
Heterotrait-Monotrait Ratio (HTMT)										
AM										
CL	0.774									
ED	0.635	0.600								
ID	0.694	0.607	0.780							
RI	0.643	0.557	0.412	0.451						
SA	0.525	0.404	0.386	0.436	0.560					
SD	0.656	0.658	0.421	0.498	0.675	0.505				
TR	0.678	0.678	0.463	0.444	0.751	0.551	0.761			
WOM	0.735	0.678	0.591	0.634	0.797	0.607	0.751	0.843		
WP	0.479	0.421	0.391	0.410	0.572	0.489	0.458	0.536	0.601	

### Structural model assessment

It is evident from the previous section that this study meets all the main criteria to measure the reliability and validity of the measurement model. Therefore, next comes the assessment of the structural model. Following the rule of thumbs, this study applies coefficient of determination (R2), a cross-validated redundancy measure (Q2). The measurement of variance also commonly known as R square (R^2^) has been estimated next to demonstrate the model's explanatory power through the endogenous variables (Shmueli and Koppius, 2011; Rigdon, 2012; Hair et al., 2019). Table 6 shows the R^2^ values, patients' satisfaction (0.336) and willingness to pay a premium (0.290) are showing a weak; whereas, repatronage intention (0.515), word-of-mouth (0.622), and patients' trust (0.565) are showing a significant level of explanatory power (Henseler et al., 2009). Finally, the Q2 values were calculated *via* the blindfolding method to estimate the predictive accuracy of the structural model (Shmueli and Koppius, 2011). Table 4 points out that repatronage intention (0.390), patients' trust (0.412), word-of-mouth (0.420), patients' satisfaction (0.225), and willingness to pay a premium (0.216) all these endogenous constructs have small to medium level of predictive relevance (Hair et al., 2019).

**Table 6:** Path coefficient.

**HT**	**Relationship**	**Beta**	**SD**	**T-statistics**	**f-square**	**R** ^2^	**Q** ^2^	**Comments**
H1a	AM → SA	0.277	0.060	4.583**	0.442	0.336	0.225	Significant
H1b	AM → TR	0.235	0.057	4.153**	0.049	0.565	0.412	Significant
H2a	ED → SA	0.050	0.065	0.778	0.052			Not Significant
H2b	ED → TR	0.080	0.057	1.416	0.307			Not Significant
H3a	ID → SA	0.118	0.060	1.967*	0.348			Significant
H3b	ID → TR	−0.110	0.054	2.039**	0.312			Significant
H4a	CL → SA	0.172	0.068	2.529**	0.443			Significant
H4b	CL → TR	0.209	0.054	3.876**	0.043			Significant
H5a	SD → SA	0.265	0.056	4.726**	0.059			Significant
H5b	SD → TR	0.445	0.051	8.792**	0.270			Significant
H6a	SA → WM	0.220	0.040	5.478**	0.094			Significant
H6b	SA → RI	0.225	0.052	4.361**	0.477			Significant
H6c	SA → WP	0.276	0.050	5.561**	0.579			Significant
H7a	SA → WM	0.653	0.037	17.583**	0.833	0.622	0.420	Significant
H7b	TR → RI	0.576	0.041	13.902**	0.505	0.515	0.390	Significant
H7c	TR -> WP	0.342	0.044	7.696**	0.122	0.290	0.216	Significant
H8	TR -> SA	0.311	0.073	4.241**	0.216			Significant

t ≥ 2.326 indicates **p <0.01 and t ≥ 1.645 indicates *p <0.05.

The hypotheses test results were obtained by running bootstrapping with the setting of, 2000 subsamples and 5,000 iterations to evaluate the structural model along with the hypotheses one by one. The standardized path coefficients obtained from bootstrapping show that ambiance had a significant positive relationship with both satisfaction (0.277; *p* < 0.01) and trust (0.235; p < 0.01), lending support to H1a and H1b. On the contrary, the path coefficients from exterior design to patients' satisfaction (0.050; p > 0.05) and patients' trust (0.080; *p* > 0.05) were positive but weak and insignificant; hence, H2a and H2b are not accepted. The path coefficients from internal decor to satisfaction (0.118; p < 0.05) and trust (-0.110; p < 0.05) had a significant relationship. Therefore, the internal decor has a significant and positive influence on patients' trust and satisfaction, thus, H3a and H3b are accepted.

**Figure 3:**
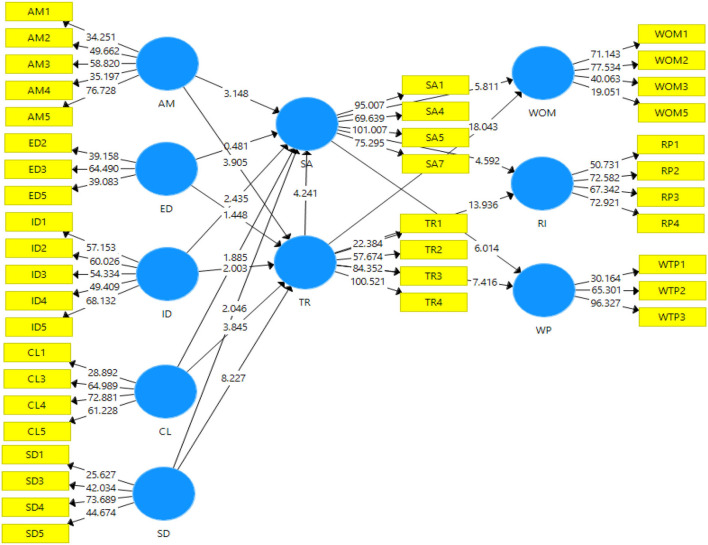
Structural model.

The path coefficient of cleanliness had a significant effect on satisfaction (0.172; *p* < 0.01) and trust (0.209; *p* < 0.01). Thus, cleanliness exhibits a positive influence on satisfaction and patients' trust, lending support to H4a and H4b. Service delivery had a positive link with satisfaction (0.265; *p* < 0.01) and trust (0.445; *p* < 0.01). Therefore, H5a and H5b are accepted. The standardized path coefficient of patients' satisfaction had a link with word-of-mouth (0.220; *p* < 0.01), repatronage intention (0.225; *p* < 0.01) and willingness to pay a premium (0.276; *p* < 0.01). Therefore, patients' satisfaction exhibits a positive and significant influence on the mentioned variables; therefore, H6a, H6b, and H6c are accepted. Patients' trust had a significant relationship with word-of-mouth (0.653; *p* < 0.01), repatronage intention (0.576; *p* < 0.01) and willingness to pay a premium (0.342; *p* < 0.01). Thus, H7a, H7b, and H7c are accepted. Figure 3 shows the structural model analysis. The results also indicated that there was a significant relationship between trust and satisfaction (0.311; *p* < 0.01), thus H8 is accepted.

Based on the report of mediating effects in Table 7, the findings revealed that satisfaction mediates the effect of ambiance, interior décor, cleanliness, and service delivery on word-of-mouth, repatronage intention, and willingness to pay a premium, therefore H9 is supported. The findings also indicated that trust mediated the effect of ambiance, interior décor, cleanliness, and service delivery on word-of-mouth, repatronage intention, and willingness to pay a premium, thus hypothesis H10 is accepted.

**Table 7:** Mediating effect.

**Relationship**	**Beta**	**SD**	**T-values**	***P*** **-values**	**Comments**
AM -> SA -> WOM	0.045	0.017	2.576	0.010	Mediating
ED -> SA -> WOM	0.006	0.012	0.460	0.646	No mediating
ID -> SA -> WOM	0.034	0.015	2.200	0.028	Mediating
CL -> SA -> WOM	−0.030	0.018	1.967	0.050	Mediating
SD -> SA -> WOM	0.028	0.016	1.788	0.050	Mediating
AM -> SA -> RI	0.046	0.020	2.336	0.020	Mediating
ED -> SA -> RI	0.006	0.013	0.455	0.649	No mediating
ID -> SA -> RI	0.034	0.016	2.083	0.038	Mediating
CL -> SA -> RI	−0.031	0.018	1.699	0.050	Mediating
SD -> SA -> RI	0.028	0.017	1.700	0.050	Mediating
AM -> SA -> WP	0.056	0.021	2.642	0.008	Mediating
ED -> SA -> WP	0.007	0.015	0.468	0.640	No mediating
ID -> SA -> WP	0.042	0.020	2.142	0.033	Mediating
CL -> SA -> WP	−0.038	0.021	1.762	0.050	Mediating
SD -> SA -> WP	0.035	0.019	1.863	0.050	Mediating
AM -> TR -> WOM	0.153	0.041	3.770	0.000	Mediating
ED -> TR -> WOM	0.052	0.037	1.425	0.155	No mediating
ID -> TR -> WOM	−0.072	0.036	2.005	0.046	Mediating
CL -> TR -> WOM	0.136	0.036	3.811	0.000	Mediating
SD -> TR -> WOM	0.291	0.039	7.441	0.000	Mediating
AM -> TR -> RI	0.135	0.038	3.606	0.000	Mediating
ED -> TR -> RI	0.046	0.032	1.446	0.149	No mediating
ID -> TR -> RI	−0.063	0.032	1.971	0.049	Mediating
CL -> TR -> RI	0.120	0.033	3.614	0.000	Mediating
SD -> TR -> RI	0.257	0.035	7.384	0.000	Mediating
AM -> TR -> WP	0.080	0.024	3.338	0.001	Mediating
ED -> TR -> WP	0.027	0.019	1.434	0.152	No mediating
ID -> TR -> WP	−0.038	0.019	1.962	0.040	Mediating
CL -> TR -> WP	0.071	0.021	3.427	0.001	Mediating
SD -> TR -> WP	0.153	0.028	5.415	0.000	Mediating

*t ≥ 2.326 considers at significant level p < 0.01 and t ≥ 1.645 considers at p < 0.05*.

## Discussion

The constructs for GP clinics' service environment consist of five variables (ambiance, interior décor, service delivery, exterior design, and cleanliness). In terms of a causal relationship between service environment variables and patients' experiences (satisfaction, and trust), ambiance, and service delivery features of GP clinics' service environment have higher associations with patients' satisfaction and trust while cleanliness and interior décor have a moderate influence. These are similar to the findings of Lee (2011) who evaluated patients' satisfaction with the outpatient clinic's physical environment, and found that the clinic's signage had a positive relationship with patients' satisfaction. Our results reaffirm Lee's as 46.9% of the respondents of this study visited the GP clinics at least three times in the past year, demonstrating the links between GP clinics' ambiance, service delivery, and patients' satisfaction.

It is deduced that the GP clinics' ambiance and service delivery are the most important and emotional responses amongst the respondents relating to the strong association. This study is also consistent with the systematic review by Han et al. (2018) which found ambiance and service delivery variables to be the most influential factors in patients' evaluation of the healthcare service environment. Lee (2011) and Laursen et al. (2014) examined hospital-based outpatient services which may be primary, or most likely specialist care services, as compared to GP settings which are primary and community-based. However, the generic setups of hospital-based outpatient and community-based GP clinics are arguably comparable. The PLS algorithm found no significant correlations between patients' satisfaction and exterior decor and trust. In addition, the findings also identified that satisfaction and trust are not mediated by the effect of exterior décor or word-of-mouth, repatronage intention, or willingness to pay a premium. This is possibly due to the patients' lower preference for exterior décor of the GP clinics, while what they want is quality healthcare delivery, ambiance, interior décor, and cleanliness from these GP clinics. Winkel and Holahan (1985) and Azila-Gbettor et al. (2013) found that external design was not significant enough as a factor to positively influence patients' viewpoint of service quality in public healthcare institutes.

The interior décor and cleanliness are significant factors that can influence patient satisfaction in hospitals. Sahoo and Ghosh (2016) also found that ambiance and service delivery are the most crucial factors influencing patients' satisfaction in a private clinic. Their study demonstrated that appealing internal decoration was a significant factor in patients' satisfaction and trust, suggestive of a change toward hedonic appreciation in a traditionally utilitarian service environment setting, particularly healthcare. Akmaz and Çadirci (2017) found that ambiance, service delivery, hygiene, and cleanliness in outpatient clinics and emergency departments had a positive impact on trust and satisfaction but external decoration did. In the private GP clinics that Ayas et al. (2008) studied, patients' perception of the waiting areas and physical surroundings was that the sitting arrangements and ambient conditions (background sound and lighting level) induced tranquility amid the waiting patients. Ambiance factors such as lighting, fragrance, and temperature serve utilitarian objectives in the healthcare service environment. These sensorial factors further have consequential impacts on the patients' satisfaction, especially when they encounter some sort of discomfort that impairs their senses. Consequently, a comfortable and peaceful ambiance would create a positive emotional experience (Lee, 2011).

Service delivery, even though itemized as a service environment factor, is not a physical stimulus in the true sense, but a social factor that is derived from the service provider's (doctor and clinic personnel) mannerisms, for example, empathy, friendliness, and kindness toward the patients. The evaluation of healthcare services relies heavily on doctor-patient interactions (Sahoo and Ghosh, 2016). Hence, it does not come as a surprise that GP clinics' service delivery positively affects patients' satisfaction. The findings highlight the significance of friendly and comfortable social interplay; for instance, the capability to convey friendliness and a sense of caring by the clinic personnel as well as the internal doctor toward patients (Akmaz and Çadirci, 2017). The findings of the study point to the links between satisfaction and word-of-mouth, and willingness to pay the premium, while the association with repatronage intention is much stronger compared to others. These outcomes are aligned with prior studies in other service sectors. For instance, Ng and Russell-Bennett (2015) stated that patients' trust and satisfaction influence their behavioral intentions such as positive word-of-mouth and repatronage intention. This is commercially beneficial from the GP clinic's business perspective, as emotionally satisfied patients are willing to pay higher fees.

## Theoretical and practical implications

The study's main theoretical contribution is the comprehensive construct of GP clinics' healthcare, which can be adopted by future healthcare studies—particularly, those intended to examine GP clinics' service environment. The findings indicated that ambiance, cleanliness, interior décor, and service delivery are the crucial factors that can increase the satisfaction and trust of patients toward healthcare services in GP clinics in Malaysia. Patients' trust is also crucial in this study because higher trust in the healthcare services can increase satisfaction, which in turn leads to word-of-mouth, repatronage intention, and willingness to pay a premium for healthcare services in the GP clinics.

This study identified four factors or antecedents that are vital to generating patients' experiences (satisfaction and trust) namely, GP clinics' ambiance, service delivery, cleanliness, and interior decor. These factors are associated with the theory of environmental psychology of the hospital (Winkel and Holahan, 1985), and servicescape. These variables can be used by researchers who intend to study GP clinics' servicescape. Researchers who intend to study other clinic-based service environments, such as outpatient clinics, specialist clinics, and dental clinics, could refer to the constructs of variables as guides.

Based on the findings, the study identified service delivery and ambiance to be the most dominant determinants for patient service evaluation of GP clinics' service environments. The findings are consistent with other healthcare studies. It also confirmed that patients see GP clinics' service environment from a utilitarian perspective. Being aligned with previous healthcare studies, this study confirms that the patients' satisfaction and trust in GP clinics' healthcare service delivery significantly influence patient behavioral response to the GP clinic.

The findings have practical implications. The results identified that service delivery reflects patients' satisfaction. The potential changing trend in patient expectation requires GP clinics to react accordingly. GP clinics must ensure optimal ambiance that is restful and comfortable. Clinical waste odors must be managed to avoid an unpleasant smell. Similarly, appropriate lighting and peaceful background sound are important. This study examined ambiance elements from utilitarian perspectives, and hedonic elements of ambient conditions such as music and fragrance could potentially provide patients with a positive experience. Perhaps, in GP clinics' service settings, the hedonic ambient elements such as calming music and fragrance that are confined to specified zones such as the waiting lounge—where no medical intervention takes place—could be of benefit. The desirable ambiance in GP clinics' waiting areas led to a sense of calm and comfort.

Service delivery is heavily dependent on the interplay between patients and service providers. That is why adequate personnel training is required in trading pleasantries and enunciating empathy and kindness. From the doctors' end, along with a sympathetic personality, doctors need to demonstrate strong medical proficiency plus the capacity to dispatch information effectively. The GP clinics' service is bound to be responsive, for instance, service providers or managers of GP clinics should assure patients' comfort at any cost, through short waiting periods and prioritize cases based on the seriousness of complaints.

In addition, the mediation analysis brings some new insights into this mechanism. Except for “exterior design” the other service environment factors namely ambiance, interior decor, cleanliness, and service delivery impacts the WOM, willingness to pay a premium, and repatronage intention when satisfaction and trust are ensured. To support this the study of Laursen et al. (2014) illustrates that the design factors might be beneficial for patients' comfort to reduce anxiety, thus, the managers must focus on these healthcare environmental design factors since they are proven to enhance greater patient satisfaction by making them feel at home. Hence, for decision-makers, it is important to design the GP clinic's service environment in such a way that must enhance the trust and patient satisfaction to make sure not only to retain the existing patients but also to attract potential ones *via* WOM. Also, as trust influences satisfaction, they should embed transparency and good governance into their service design which in turn reflects trustworthiness.

## Conclusion

The study has made a significant contribution to the healthcare service settings that vary across service types (e.g., primary care, specialist cares), service location (e.g., community-based, hospital-based), the field of specializations, and intensive care units. Because of the differences in physical settings, researchers customized their service environment constructs to suit particular healthcare types of interest. This study has comprehensively categorized and classified different physical features and elements of GP clinics' service environment. The findings indicate that a GP clinic's exterior, space, layout, decoration, ambiance, cleanliness, and service delivery are crucial components that increase patients' satisfaction and trust in healthcare services at GP clinics. These findings can be used by researchers who intend to study GP clinics' servicescape. Researchers who intend to study other clinic-based service environments, such as outpatient clinics, specialist clinics, and dental clinics, could refer to the findings as guides.

The findings also indicate that ambient and service delivery are the most crucial factors for patients in healthcare service evaluation of a GP clinic's service environment. The findings are consistent with other healthcare studies. The study reveals that patients' satisfaction with the GP clinic's physical environment significantly influences their behavioral response in positive word-of-mouth, willingness to pay a premium, and repatronage intention. In the GP clinics' healthcare service environment, ambiance, service delivery, interior décor, and cleanliness are found to be influential toward patients' satisfaction and trust. These findings are expected to assist general practice hospitals to reform or even transform their service setting to cater to particular market segments.

In the domain of healthcare studies, it was repeatedly established that ambiance acts as a major influencer for the trust and satisfaction of the patient from a utilitarian viewpoint. Exterior decor does not have significant relationships with satisfaction and trust. The ambiance and service delivery should be optimized by GP clinics. The evaluation of healthcare services is highly dependent on the patients' experience and emotions during the service encounter. Therefore, the performance of healthcare service providers is highly reliant on the patients' experience during the service encounter. High-quality healthcare services increase patient satisfaction while poor-quality healthcare services lead to dissatisfaction. The healthcare service environment reflects patients' experiences which in turn influence their behavior. In the GP clinic service environment, ambiance, space, function, signs, and symbols are crucial for patients' satisfaction with healthcare services.

The limitation of this study includes memory-based data collection, the inclusion of respondents visiting GP services based on panel clinics, and uneven locational distribution of GP clinics. Future healthcare studies are recommended to address these issues. The respondents of this study were patients in Malaysia and thus results of this study cannot be generalized to other countries. Future research can be conducted on healthcare costs for medical treatments, and explore the factors that affect the quality of healthcare services in other countries. The internal and external factors related to the healthcare services such as availability of resources for healthcare services, physicians and nurses' cooperation, patients' cooperation, and collaboration among healthcare service providers affect the healthcare quality services and patient outcomes. Furthermore, studies on specific demographics may allow GP clinics to cater to specific market segments. As service delivery is a significant factor, detailed studies of the service quality aspect are recommended. The changing nature of primary care service delivery necessitates future studies to investigate the benefits of alternative primary care options.

## Data availability statement

The original contributions presented in the study are included in the article/Supplementary material, further inquiries can be directed to the corresponding authors.

## Ethics statement

Ethical review and approval was not required for the study on human participants in accordance with the local legislation and institutional requirements. Written informed consent from the patients/participants was not required to participate in this study in accordance with the national legislation and the institutional requirements.

## Author contributions

YA, MKR, and MN designed the overall framework of the research and drafted the manuscript. MG and MAR conducted the literature review. AM and XC reviewed the draft. All authors read the final manuscript and approved it for final submission.

## Funding

This study was supported by the Key Planning Project of Educational Science of Jiangxi Province (Grant 17ZD059).

## Conflict of interest

The authors declare that the research was conducted in the absence of any commercial or financial relationships that could be construed as a potential conflict of interest.

## Publisher's note

All claims expressed in this article are solely those of the authors and do not necessarily represent those of their affiliated organizations, or those of the publisher, the editors and the reviewers. Any product that may be evaluated in this article, or claim that may be made by its manufacturer, is not guaranteed or endorsed by the publisher.
