# Supplementary material for: Determinants of patients' satisfaction and trust toward healthcare service environment in general practice clinics
Source: Front Psychol. 2022 Jul 29;13:856750. doi: 10.3389/fpsyg.2022.856750 (PMC9373924; doi:10.3389/fpsyg.2022.856750)
Supplement: Supplementary file 1 [file Data_Sheet_1.docx]

Survey Questionnaire:

| SECTION A: PRIVATE GP CLINIC INFORMATION |
| --- |

Think about your **LAST VISIT** to a **PRIVATE GP CLINIC** for a health issue or medical service. How would you rate the following questions?

| **1. Name of the GP clinic you last visited:**  ......................................... (Optional) | **2. What is the type of your GP clinic?**   - Chain Clinics e.g. Mediveron, Qualitas - Sole Proprietor e.g. Klinik Dr. Tey - Others, please specify:   ………………….………. |
| --- | --- |
| **3. Where is the GP clinic located?**   - City centre - Suburb/ residential area - Rural area | **4. How long have you been visiting the GP clinic?**   - < 6 months - 6 months – 1 year - 1 – 3 years - 3 – 5 years - > 5 years |
| **5. You chose the GP clinic because... 󠅊**   - Close to home/ residence - The GP has a good reputation - The GP is my family doctor - Company’s panel clinic - Insurance’s panel clinic - Others, please specify:   ………………….………. | **6. The most common reason for your GP clinic visit?**   - Mild illness e.g. common cough and cold - Longstanding illness e.g diabetes - Beauty/ aesthetics - Pregnancy-related - Companionship/ counselling - Others, please specify:   ………………….………. |
| **7. In the past year, how many times have you visited the GP clinic?**   - 1 - 2 times - 3 - 4 times - 5 - 6 times - > 6 times | **8. In general, how would you rate your overall health?**   - Poor - Fair - Good - Very Good - Excellent |

| **SECTION B: THE ENVIRONMENT OF GP CLINIC** |
| --- |

Please indicate your level of agreement on each of the following statements about your **EXPERIENCE** visiting the **GP clinic you mentioned above**

|  | **Clinic Exterior** | **Strongly Disagree** | **Disagree** | | **Neutral** | | **Agree** | **Strongly Agree** |
| --- | --- | --- | --- | --- | --- | --- | --- | --- |
| 1. | The clinic was visible from a distance | 1 | | 2 | 3 | 4 | | 5 |
| 2. | The building in which the clinic was located is distinguishable | 1 | | 2 | 3 | 4 | | 5 |
| 3. | The clinic was conveniently located | 1 | | 2 | 3 | 4 | | 5 |
| 4. | I could find a parking space easily | 1 | | 2 | 3 | 4 | | 5 |
| 5. | The clinic signage board was eye-catching | 1 | | 2 | 3 | 4 | | 5 |
| 6. | The clinic name was appealing | 1 | | 2 | 3 | 4 | | 5 |
| 7. | The clinic exterior (e.g. entrance, glass panel) was well-designed | 1 | | 2 | 3 | 4 | | 5 |
| 8. | The entrance was accessible | 1 | | 2 | 3 | 4 | | 5 |

|  | **Clinic Interior** | **Strongly Disagree** | **Disagree** | **Neutral** | **Agree** | **Strongly Agree** |
| --- | --- | --- | --- | --- | --- | --- |
| 1. | The registration counter was well-placed | 1 | 2 | 3 | 4 | 5 |
| 2. | The pharmacy counter was well-positioned | 1 | 2 | 3 | 4 | 5 |
| 3. | The waiting area was spacious | 1 | 2 | 3 | 4 | 5 |
| 4. | The aisles/ corridors were wide | 1 | 2 | 3 | 4 | 5 |
| 5. | The flooring was attractive | 1 | 2 | 3 | 4 | 5 |
| 6. | The wall colour/ wall paper design was pleasing | 1 | 2 | 3 | 4 | 5 |
| 7. | The displays (e.g. paintings, pictures, posters) were aesthetic | 1 | 2 | 3 | 4 | 5 |
| 8. | The furniture was stylish | 1 | 2 | 3 | 4 | 5 |
| 9. | The consultation room was accessible | 1 | 2 | 3 | 4 | 5 |
| 10. | The consultation room was large | 1 | 2 | 3 | 4 | 5 |
| 11. | In general, the clinic size was adequate | 1 | 2 | 3 | 4 | 5 |
| 12. | The clinic’s layout was easy to navigate/ move around | 1 | 2 | 3 | 4 | 5 |
| 13. | In general, the clinic decoration was attractive | 1 | 2 | 3 | 4 | 5 |

|  | **Clinic Ambience** | **Strongly Disagree** | **Disagree** | **Neutral** | **Agree** | **Strongly Agree** |
| --- | --- | --- | --- | --- | --- | --- |
| 1. | The temperature was acceptable | 1 | 2 | 3 | 4 | 5 |
| 2. | The lighting was relaxing | 1 | 2 | 3 | 4 | 5 |
| 3. | The clinic scent/ smell was pleasant | 1 | 2 | 3 | 4 | 5 |
| 4. | The surrounding sound was peaceful | 1 | 2 | 3 | 4 | 5 |
| 5. | In general, the clinic ambience was comforting | 1 | 2 | 3 | 4 | 5 |

|  | **Cleanliness** | **Strongly Disagree** | **Disagree** | **Neutral** | **Agree** | **Strongly Agree** |
| --- | --- | --- | --- | --- | --- | --- |
| 1. | The sidewalk in front of the clinic was clean | 1 | 2 | 3 | 4 | 5 |
| 2. | The reception area was clean | 1 | 2 | 3 | 4 | 5 |
| 3. | The waiting area was clean | 1 | 2 | 3 | 4 | 5 |
| 4. | The consultation room was clean | 1 | 2 | 3 | 4 | 5 |
| 5. | The staff's attire were clean | 1 | 2 | 3 | 4 | 5 |

|  | **Service Delivery** | **Strongly Disagree** | **Disagree** | **Neutral** | **Agree** | **Strongly Agree** |
| --- | --- | --- | --- | --- | --- | --- |
| 1. | The registration personnel was friendly | 1 | 2 | 3 | 4 | 5 |
| 2. | I did not have to wait long to see the doctor | 1 | 2 | 3 | 4 | 5 |
| 3. | The doctor’s explanation was clear | 1 | 2 | 3 | 4 | 5 |
| 4. | The doctor was caring towards me | 1 | 2 | 3 | 4 | 5 |
| 5. | The assisting nurse was kind to me | 1 | 2 | 3 | 4 | 5 |

**SECTION C: OVERALL EXPERIENCE WITH THE GP CLINIC**

Please indicate your level of agreement on each of the following statements about your **EXPERIENCE** visiting the **GP clinic you mentioned above**

|  |  | **Strongly Disagree** | **Disagree** | **Neutral** | **Agree** | **Strongly Agree** |
| --- | --- | --- | --- | --- | --- | --- |
| 1. | I am sure that my personal information is kept confidential by the clinic | 1 | 2 | 3 | 4 | 5 |
| 2. | I am confident with the clinic | 1 | 2 | 3 | 4 | 5 |
| 3. | I expect the clinic to deliver its best | 1 | 2 | 3 | 4 | 5 |
| 4. | I trust the clinic | 1 | 2 | 3 | 4 | 5 |

Please indicate your level of agreement on each of the following statements about your **INTENTION** after visiting the **GP clinic you mentioned above**

|  |  | **Strongly Disagree** | **Disagree** | **Neutral** | **Agree** | **Strongly Agree** |
| --- | --- | --- | --- | --- | --- | --- |
| 1. | I will say positive things about the clinic to others | 1 | 2 | 3 | 4 | 5 |
| 2. | I will encourage friends/ relatives to go to the clinic | 1 | 2 | 3 | 4 | 5 |
| 3. | I will give positive reviews of the clinic on social media | 1 | 2 | 3 | 4 | 5 |
| 4. | I will use social media to promote the clinic to friends/ relatives | 1 | 2 | 3 | 4 | 5 |
| 5. | I will recommend the clinic to others on social media | 1 | 2 | 3 | 4 | 5 |

|  |  | **Strongly Disagree** | **Disagree** | **Neutral** | **Agree** | **Strongly Agree** |
| --- | --- | --- | --- | --- | --- | --- |
| 1. | I don’t mind paying extra for a reputable clinic | 1 | 2 | 3 | 4 | 5 |
| 2. | I am willing to pay an expensive fee for this clinic | 1 | 2 | 3 | 4 | 5 |
| 3. | I will pay more for this clinic than other GP clinics | 1 | 2 | 3 | 4 | 5 |

|  |  | **Strongly Disagree** | **Disagree** | **Neutral** | **Agree** | **Strongly Agree** |
| --- | --- | --- | --- | --- | --- | --- |
| 1. | I am likely to visit this clinic in future | 1 | 2 | 3 | 4 | 5 |
| 2. | I see myself revisiting this clinic for my next health check-up | 1 | 2 | 3 | 4 | 5 |
| 3. | This clinic will be my first choice for my next health examination | 1 | 2 | 3 | 4 | 5 |
| 4. | I have every intention of visiting this clinic in future | 1 | 2 | 3 | 4 | 5 |

The following words concern your **FEELINGS** about your GP Clinic. **Select the number that best indicates** how you feel about your GP Clinic

| HAPPY | 1 | 2 | 3 | 4 | 5 | UNHAPPY |
| --- | --- | --- | --- | --- | --- | --- |
| PLEASED | 1 | 2 | 3 | 4 | 5 | DISPLEASED |
| ENJOYABLE | 1 | 2 | 3 | 4 | 5 | FRUSTRATING |
| SATISFIED | 1 | 2 | 3 | 4 | 5 | UNSATISFIED |
| RESPECTED | 1 | 2 | 3 | 4 | 5 | DISRESPECTED |
| WELCOMED | 1 | 2 | 3 | 4 | 5 | UNWELCOMED |

| **SECTION D : DEMOGRAPHIC INFORMATION** |
| --- |

| **1. Gender**   - Male - Female | **3. Age**   - 20 years old and below - 21-30 years old - 31-40 years old - 41-50 years old - 51-60 years old - 61 years old and above |
| --- | --- |
| **2. Ethnicity**   - Malay - Chinese - Indian - Others, Please Specify: ……….. |  |
| **4. Marital Status**   - Single - Married - Widowed - Divorced | **5. Level of Education**   - High School - Professional Certification - Bachelor’s Degree - Master’s Degree - Doctorate |
| **6. Occupation**   - Unemployed - Student - Self-employed - Government employee - Private employee | **7. Your Monthly Income**   - None - Up to RM 2,500 - Up to RM 5,000 - Up to RM 10,000 - Above RM 10,000 |
